# Longitudinal Associations Between Hearing Loss and General Cognitive Ability: The Lothian Birth Cohort 1936

**DOI:** 10.1037/pag0000385

**Published:** 2019-08-08

**Authors:** Judith A. Okely, Michael A. Akeroyd, Michael Allerhand, John M. Starr, Ian J. Deary

**Affiliations:** 1 Centre for Cognitive Ageing and Cognitive Epidemiology, Department of Psychology, University of Edinburgh; 2 Hearing Sciences, Division of Clinical Neurosciences, School of Medicine, University of Nottingham; 3 Centre for Cognitive Ageing and Cognitive Epidemiology, Department of Psychology, University of Edinburgh

**Keywords:** cognitive ageing, hearing loss, childhood cognitive ability, longitudinal study

## Abstract

Hearing impairment is associated with poorer cognitive function in later life. We tested for the potential contribution of childhood cognitive ability to this relationship. Childhood cognitive ability is strongly related to cognitive function in older age, and may be related to auditory function through its association with hearing impairment risk factors. Using data from the Lothian Birth Cohort, 1936, we tested whether childhood cognitive ability predicted later-life hearing ability then whether this association was mediated by demographic or health differences. We found that childhood cognitive ability was negatively associated with hearing impairment risk at age 76 (odds ratio = .834, *p* = .042). However, this association was nonsignificant after subsequent adjustment for potentially mediating demographic and health factors. Next, we tested whether associations observed in older age between hearing impairment and general cognitive ability level or change were accounted for by childhood cognitive ability. At age 76, in the minimally adjusted model, hearing impairment was associated with poorer general cognitive ability level (β = −.119, *p* = .030) but was not related to decline in general cognitive ability. The former association became nonsignificant after additional adjustment for childhood cognitive ability (β = −.068, *p* = .426) suggesting that childhood cognitive ability contributes (potentially via demographic and health differences) to the association between levels of hearing and cognitive function in older age. Further work is needed to test whether early life cognitive ability also contributes to the association (documented in previous studies) between older-age hearing impairment and cognitive decline.

Advancing age is associated with an increased risk of cognitive decline, a condition that affects independence, and is becoming an increasing social and financial burden as a result of the ageing global population (Abrahamson, Clark, Perkins, & Arling, 2012; Deary, Corley, et al., 2009; Tucker-Drob, 2011b). Older age is also associated with a higher risk of hearing impairment, with 63% of Americans aged 70 and older experiencing hearing losses of at least mild severity (Bainbridge & Wallhagen, 2014). Recently, the relationship between declining cognitive and auditory health has attracted increased attention, and findings from observational studies have highlighted the possibility that hearing impairment is a risk factor for age-related cognitive impairment and decline (Loughrey, Kelly, Kelley, Brennan, & Lawlor, 2018).

Although hearing ability declines steadily with age there is substantial variation in terms of onset and progression of age-related hearing impairment (Davis, 1995). This condition can be accelerated by noise exposure (Lie et al., 2016; D. I. Nelson, Nelson, Concha-Barrientos, & Fingerhut, 2005), lifestyle factors including smoking (Gopinath et al., 2010; Nomura, Nakao, & Morimoto, 2005), and chronic health conditions that include diabetes (Fowler & Jones, 1999), and cardiovascular disease (Gates, Cobb, D’Agostino, & Wolf, 1993). Hearing loss is more common among men than women (Stevens et al., 2013); however, this sex difference may be explained by the higher prevalence of other hearing loss risk factors (e.g., noise exposure) in men (Helzner et al., 2005).

A key finding in the field of intelligence research is that individuals who perform well on one mental task are likely to do well on other tasks too, irrespective of the type of mental processes involved. This phenomenon is described by the construct of general cognitive ability (Spearman, 1904). General cognitive ability is typically modeled as the pinnacle of a hierarchical structure below which are domain-specific abilities (abilities grouped according to cognitive ability domain) and, below that, test-specific abilities (abilities not accounted for by general or domain specific abilities; Carroll, 1993; see Deary, 2013; Figure 1). General cognitive ability is often operationalized as the first unrotated component in a principal component analysis of several cognitive tests, and will typically account for about 40% of the total variance in tests’ scores. A general factor appears to account for a substantial proportion of between-person differences in age-related cognitive decline too. In a study using data collected at ages 70, 73, and 76 from the Lothian Birth Cohort 1936, 48% of between-person differences in cognitive decline was shared across 13 different mental ability tests (Ritchie et al., 2016). A similar general factor of cognitive decline was found by Ghisletta, Rabbitt, Lunn, and Lindenberger (2012) and Tucker-Drob (2011a). In addition to declining general cognitive ability, the ageing process highlights two distinct domains of cognitive function: crystallized ability (learned knowledge and experience) and fluid ability (the use of deliberate reasoning to perceive conceptual relationships and solve novel problems; Cattell, 1943, 1963). Although they are strongly correlated, these domains of cognitive ability are characterized by distinct age-related trajectories, with crystallized ability increasing up to the age of 60, with only slight decline thereafter (Hedden & Gabrieli, 2004), and fluid ability declining from early adulthood onward (Horn & Cattell, 1967).

A recent meta-analysis of 40 studies examining the association between hearing impairment and cognitive function, cognitive impairment or dementia risk, found evidence of a small but significant association between hearing impairment and all three of these cognitive outcomes (Loughrey et al., 2018). Among cross-sectional studies, the pooled correlation coefficient for the association between hearing impairment and cognitive abilities was *r* = −.12, 95% confidence interval (CI) [−.14, −.10]. Among prospective studies, which had a follow up ranging from 2 to 23 years (*M* = 10.4, *SD* = 6.7 years) the pooled correlation between hearing impairment and subsequent cognitive function was *r* = −.09, 95% CI [−.11, −.07]. Some studies included in the meta-analysis additionally tested for correlated changes in hearing and cognitive abilities with ageing. Two reported a correlation between declining cognitive and hearing abilities (Anstey, Hofer, & Luszcz, 2003; Valentijn et al., 2005), and one study found no such effect (Anstey, Luszcz, & Sanchez, 2001).

The association between hearing loss and dementia risk was further highlighted in the Lancet Commission on Dementia Prevention, Intervention, and Care (Livingston et al., 2017). Based on a meta-analysis of three longitudinal studies of initially healthy people, the Lancet Commission argued that elimination of midlife hearing loss could result in a nine percent reduction in new cases of dementia. This estimate placed midlife hearing loss as the most influential potentially modifiable risk factor for dementia compared with eight other lifestyle and health factors, including smoking, obesity, and fewer years of education (Livingston et al., 2017).

Previous work illustrates the link between declining auditory and cognitive health, however, it is still largely unclear how these two processes are related; although, potential underlying mechanisms have been proposed (Lindenberger & Baltes, 1994). Lin et al. (2011, 2013) suggest that hearing impairment may play a causal role in the development of cognitive decline. The “sensory deprivation” hypothesis, which provides one possible explanation for such a link, suggests that reduced sensory stimulation caused by a hearing impairment limits opportunities for intellectual stimulation, which over the longer term, leads to poorer cognitive functioning (Lindenberger & Baltes, 1994). Another view, termed the “effortfulness” or “information degradation” hypothesis, is that in individuals with a hearing impairment, cognitive resources are reallocated to the processing of auditory information to the detriment of other cognitive functions (Lindenberger & Baltes, 1994; McCoy et al., 2005). Whereas the sensory deprivation model indicates that hearing impairment can lead to permanent declines in cognitive function, the effortfulness hypothesis emphasizes that changes in cognitive performance are reversible, for instance by the introduction of hearing aids (Lindenberger & Baltes, 1994; Wayne & Johnsrude, 2015). Others have suggested that sensory impairment-cognitive decline associations are caused by a third factor that impacts both auditory and cognitive health in later life (Lindenberger & Baltes, 1994; Tobias et al., 1988). Several factors that may underlie such a common cause association have been identified, and include cerebrovascular disease, social factors (such as education), and genetics (Kiely, Gopinath, Mitchell, Luszcz, & Anstey, 2012; Kuo et al., 2005; Kurniawan et al., 2012; Lin & Albert, 2014; Small, Rosnick, Fratiglioni, & Bäckman, 2004).

The theories outlined above describe how hearing and cognitive abilities might become interrelated in older age; here we take advantage of an unusually informative dataset, going back nearly 75 years, to test the possibility that associations between hearing and cognitive function, observed in older age, originate at an earlier stage in the life-course. Specifically, we hypothesized that childhood cognitive ability might account for associations between auditory and cognitive health observed in older age. Such an effect would be expected if childhood cognitive ability is related to older-age cognitive ability and older-age hearing impairment. The link between childhood cognitive ability and older-age cognitive ability is already established. Previous longitudinal work shows that cognitive ability is a stable trait, with cognitive function in childhood accounting for around 50% of the variance in cognitive ability in older age (Deary, Whalley, & Starr, 2009). We are not aware of any previous work documenting a link between childhood cognitive ability and risk of hearing impairment in older-age. However, we predicted such an association for two reasons. First, individuals with a higher cognitive ability in childhood are less likely to engage in health harming behaviors (including smoking) and have a lower risk of chronic conditions in midlife (Batty, Deary, & Macintyre, 2007; Singh-Manoux et al., 2009; Wraw, Deary, Gale, & Der, 2015; Wraw, Der, Gale, & Deary, 2018); these health factors could in turn reduce the risk of hearing impairment in older age. Second, an association between hearing acuity and cognitive function has been observed in samples of children as young as 4 years old, and in middle aged adults (Li, Jordanova, & Lindenberger, 1998; Rönnberg, Hygge, Keidser, & Rudner, 2014). Therefore, hearing-cognition associations observed in older populations may potentially originate in childhood. Such lifelong shared variance between cognitive and hearing abilities, would be consistent with the “system integrity” hypothesis, that there is a latent trait of “optimal bodily functioning” that originates early in life and accounts for shared variance in different functions across the life course (Deary, 2012).

Two mechanisms outlined above describe different potential roles of childhood cognitive ability with respect to hearing capability in older age: the first identifies childhood cognitive ability as a potential predictor of lifestyle and/or environmental factors that, in turn, are related to hearing impairment risk; and the latter suggests that childhood cognitive ability is a marker of initial “system integrity” (Deary, 2012), a latent trait that is associated with early life cognitive ability and sensory function, as well as diverse other aspects of good health. These two potential mechanisms are not mutually exclusive: individuals with greater system integrity and, thus, higher cognitive and hearing abilities may also engage in more protective health behaviors and be exposed to safer environments in adulthood. However, we emphasize that: the first idea is a life-course model in which childhood cognitive function has later indirect health consequences; and the second, system integrity idea posits individual differences in general bodily wellbeing from youth that track to a detectable extent across the life course.

The Lothian Birth Cohort 1936 (LBC1936) is a narrow-age cohort study of participants all born in 1936. Participants have completed cognitive and other testing on a triennial basis since the age of 70 (Deary, Gow, Pattie, & Starr, 2012; Deary et al., 2007; Taylor, Pattie, & Deary, 2018). Unusually, valid data on cognitive ability at age 11 are also available. Hearing impairment was assessed at ages 76 and 79, making it possible to model the association between hearing impairment and general cognitive ability level at age 76 and also with change over the subsequent 3 years. LBC1936 data, thus, provide a rare opportunity to test: (a) whether childhood cognitive ability is related to risk of later-life hearing impairment; (b) whether any such association is accounted for by demographic or health differences; and (c) whether childhood cognitive ability contributes to the association between hearing impairment and general cognitive ability level or change in older age. Note that we could not fully test the system integrity hypothesis as data on childhood hearing ability were not collected.

## Method

### Participants

The LBC1936 is a narrow-age cohort study of individuals born in 1936, and mostly living in the Edinburgh and Lothians areas of Scotland when contacted and recruited in older age. Most LBC1936 participants also took part in an earlier study (the SMS1947) that tested the mental ability of 70,805 Scottish schoolchildren born in 1936 at a mean age of 11 years (Scottish Council for Research in Education, 1949). Therefore, data on most LBC1936 participants’ IQ at age 11 is also available. Wave 1 of the LBC1936 study took place between 2004 and 2007, with 1,091 participants, at a mean age of 70 years (Deary et al., 2012, 2007; Taylor et al., 2018). Subsequent waves of LBC1936 testing were conducted on a triennial basis, with Waves 2, 3, and 4 taking place between 2007 and 2010, 2011–2013, and 2014–2017, respectively. Ethical approval was obtained from the Multi-Centre Ethics Committee for Scotland and the Lothian Research Ethics Committee. All participants provided written informed consent.

Objectively measured hearing acuity was first assessed at Wave 3, and again at Wave 4; therefore, we used data from these two waves in the current analysis. 697 participants (mean age = 76, *SD* = .68) attended Wave 3, and 550 participants (mean age = 79, *SD* = .62) attended Wave 4.

Participants who attended Wave 3 of testing (at age 76) were eligible for inclusion in the analytical sample. Of the 697 participants who attended, we excluded one participant because of childhood-onset hearing loss. As the focus of the present study was on the potential association between hearing impairment and nonpathological age-related cognitive decline, we further excluded 23 participants with possible dementia (as indicated by a Mini Mental State Examination score of less than 24 at age 76 or age 79) from the analysis predicting cognitive ability level and change. These exclusions resulted in an analytical sample of 696 for analysis predicting hearing impairment and 673 for analysis predicting older-age cognitive ability level and change.

Table 1 shows the characteristics of the analytical sample at ages 76 and 79, along with the number of participants with available data on each of the hearing, cognitive, and covariate variables at each wave. Of the participants included in the analytical sample, 42 did not have age 11 IQ scores available. These data were missing if participants were absent from school on the day of testing or attended a private school that did not administer the test. In the analytical sample, participants missing age 11 IQ scores did not differ from participants with available age 11 IQ scores on any of the demographic, health or hearing variables reported in this study. Performance on the cognitive ability tests, administered at age 76, also did not differ between participants with and without age 11 IQ scores.[Table-anchor tbl1]

Online supplemental material Table 1 shows differences at age 76 between participants who provided data at ages 76 and 79 (‘completers’) and participants who provided data at age 76 only (‘noncompleters’). Completers performed better than noncompleters on all the cognitive tests at age 76. Completers were also more likely to be nonsmokers, be from a higher occupational social class, have a higher age 11 IQ, and a lower HADS score. There were no significant differences between completers and noncompleters in terms of hearing impairment, sex, or history of chronic disease.

LBC1936 participants who left the study before age 76 (Wave 3) on average achieved lower scores on the age 11 IQ test and lower scores on the MMSE at age 70 (Wave 1). They were more likely to report a history of stroke but not diabetes, CVD, or hypertension at age 70. Further information regarding attrition between waves 1 and 4 of the LBC1936, and how attrition affects key variables at each wave can be found in the Lothian Birth Cohorts profile update paper (Taylor et al., 2018).

### Measures

#### Cognitive ability at ages 76 and 79

The same battery of 13 cognitive tests was administered to participants at each wave of the study. These were the Spatial Span (Forward and Backward) subtest from the Wechsler Memory Scale-Third UK Edition (Wechsler, 1998b); the Matrix Reasoning and Block Design subtests from the from the Wechsler Adult Intelligence Scale-Third UK Edition (Wechsler, 1998a); the Symbol Search and Digit-Symbol Substitution tests from the Wechsler Adult Intelligence Scale-Third UK Edition (Wechsler, 1998a); a computer-based inspection time test (Deary et al., 2004); and a four-choice reaction time (RT) test (Crawford, Stewart, Garthwaite, Parker, & Besson, 1988; Deary, Der, & Ford, 2001); the Digit Span Backward subtest from the Wechsler Adult Intelligence Scale-Third UK Edition (Wechsler, 1998a); the Verbal Paired Associates and Logical Memory subtests from the Wechsler Memory Scale-Third UK Edition (Wechsler, 1998b); the National Adult Reading Test (NART; Nelson & Willison, 1991); the Wechsler Test of Adult Reading (Wechsler, 2001); and a test of phonemic verbal fluency (Lezak, 2004). Internal consistency and other psychometric data for these tests is documented in detail elsewhere (Crawford et al., 1988; Deary et al., 2004; H. E. Nelson & Willison, 1991; The Psychological Corporation, 1997).

Participants also completed the Mini Mental State Examination (MMSE; Folstein, Folstein, & McHugh, 1975), which is a dementia screening instrument. Possible scores range from 0 to 30, with a score of less than 24 sometimes used to indicate possible cognitive impairment (Tombaugh & McIntyre, 1992).

#### Cognitive ability at age 11

At age 11, LBC1936 participants completed the Moray House Test No. 12. The test consists of 71 items (with a maximum score of 76) that mainly assess verbal reasoning but also includes items related to arithmetic, visuospatial ability, and cypher coding (Scottish Council for Research in Education, 1949). Participants’ scores were corrected for age in days at time of testing.

#### Hearing acuity

Hearing acuity was assessed with the Siemens HearCheck Navigator (Parving, Sørup Sørensen, Christensen, & Davis, 2008). In two evaluation studies, this hand-held screening device was found to have good sensitivity (ranging from 78 to 92%) and acceptable or good specificity (ranging from 65 to 95%) when compared with results from pure tone audiometry (Fellizar-Lopez et al., 2011; Parving et al., 2008). Data on test–retest reliability of the Siemens HearCheck Navigator was not reported in these studies; however, Fellizar-Lopez et al. (2011) report that levels of accuracy, sensitivity and specificity were consistent across two measurement occasions (inside a soundproof booth and in a quiet room). Participants who attended cognitive and physical testing at age 76 and 79 were invited to take part in the hearing acuity test. Hearing aids, if worn, were removed for the test. The HearCheck screener produces three pure high-frequency tones at 3 kHz (kHz) at decreasing intensities of 75, 55, and 35 dB Hearing Level (dB HL), and three pure midfrequency tones at 1 kHz at decreasing intensities of 55, 35, and 20 dB HL. Participants were instructed to raise their hand each time they heard a tone. This test was conducted for both ears in turn. Data used to categorize hearing impairment was taken from trials with the best hearing ear. As has been done previously with HearCheck data (Lassale, Batty, Steptoe, & Zaninotto, 2017), participants who missed at least one of the six tones (the maximum possible) were categorized as hearing impaired. Participants with hearing impairment were further categorized as having a mild impairment if they missed one tone at either 1 or 3 kHz, and as having a moderate hearing impairment if they missed two tones at either 1 or 3 kHz. Participants who missed all three tones at either 1 or 3 kHz were categorized as having a severe hearing impairment. Our conversion from number of tones heard to category of hearing loss was based on analysis of audiometric prevalence data from the U.K. National Study of Hearing (Davis, 1995, Table 109). Parametric curves were fitted to the 10, 20, 50, 80, and 90% percentiles of hearing loss at each frequency for people aged 71–80 in their better ear. We used these curves to interpolate the transformation from by-frequency hearing loss (i.e., the audiogram, determining the number of tones heard) to across-frequency hearing loss (i.e., the average hearing loss, defining the categories) while matching the population profile of both. We found that a simple conversion rule (0 misses = normal, 1 miss = mild, 2 misses = moderate, 3 misses = severe) gave a match to the World Health Organization category boundary for normal to mild hearing loss and was only slightly over (3 dB) for the mild to moderate boundary (Mathers, Smith, & Concha, 2000); thus, being sufficiently close for our analyses.

Because of the low number of participants with a severe hearing impairment (10 participants at age 76 and 7 participants at age 79), this category was grouped together with moderate hearing impairment for the purposes of the analysis. Hearing aid use was also recorded: participants were asked to report (yes or no) whether they usually wore a hearing aid.

Participants completed the cognitive and the hearing acuity tests at the Wellcome Trust Clinical Research Facility at the Western General Hospital, Edinburgh. All tests were conducted in a quiet room at the clinic where ambient environmental sound was kept to a minimum.

#### Covariates

In addition to childhood cognitive ability (age 11 IQ), we adjusted for the potential effect of age, sex, occupational social class, symptoms of anxiety and depression, smoking status, and history of diabetes (Type I or 2), cardiovascular disease, stroke, or hypertension. These factors have previously been associated with age-related hearing impairment (Heine & Browning, 2002; Huang & Tang, 2010; Kramer, Kapteyn, Kuik, & Deeg, 2002) and cognitive ability in older age (Bierman, Comijs, Jonker, & Beekman, 2007; Corley, Cox, & Deary, 2018; Plassman, Williams, Burke, Holsinger, & Benjamin, 2010; Snyder et al., 2015). In addition to potentially confounding or mediating hearing-cognition associations, history of chronic disease could reflect the effect of “a common cause” that impacts risk of hearing impairment, cognitive decline and chronic disease. We also controlled for hearing aid use, as this factor might moderate associations between hearing impairment and cognitive health. A previous study found that, among people with a hearing impairment, hearing aid users experienced less severe declines in cognitive ability (Deal et al., 2015). We note that this variable could act as a proxy of hearing impairment (i.e., only hearing impaired individuals will wear a hearing aid) and may, therefore, control for some variance in cognitive function related to hearing impairment (rather than hearing aid use).

Participants were asked to report their “main occupation” for the occupational social class variable, which was indexed according to six categories, ranging from professional to unskilled, following the Classifications of Occupations system (General, 1991). Married women whose spouse had a higher occupational social class than their own, were assigned to the higher class. Symptoms of depression and anxiety were assessed with the 14 item Hospital Anxiety and Depression scale (HADS; Zigmond & Snaith, 1983). Possible scores for this scale range from 0 to 42, with higher scores indicating higher levels of distress. Participants self-reported whether they had ever been diagnosed with diabetes, cardiovascular disease, stroke, or hypertension. Age in days, symptoms of anxiety and depression, smoking status, and chronic disease history were recorded at Waves 3 and 4, and treated as time-varying covariates in the analysis.

Corrected and uncorrected visual acuity in the right and left eyes was recorded at age 76 and 79 using a Snellen-type chart. Although visual acuity was not included in the main analysis, at the request of a referee, we tested for an association between hearing impairment and uncorrected visual acuity (in the better-seeing eye). For statistical analysis, the Snellen fraction was converted to logMAR (logarithm of the minimum angle of resolution). Partially complete lines were handled by rounding up to the previous line if the participant missed more than half of the letters. If the participant identified half or more than half of the letters on a particular line they were scored as if they had read the whole line.

### Analysis

**Figure 1 fig1:**
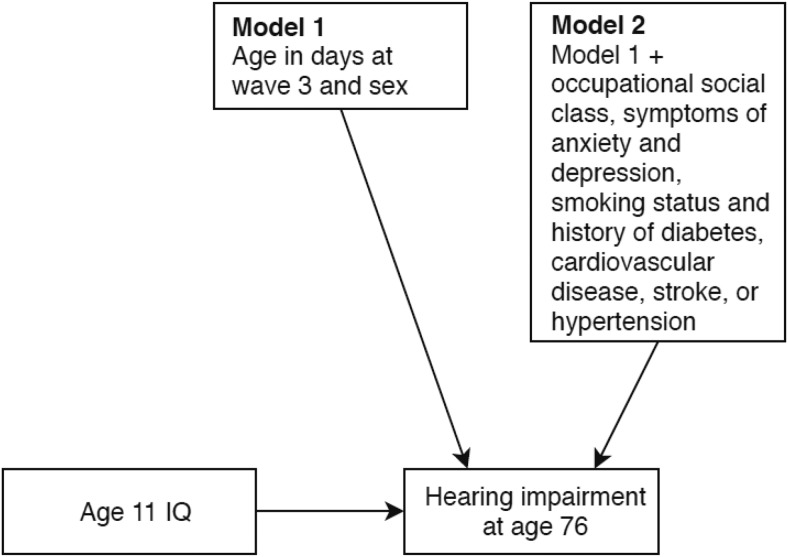
Ordinal logistic regression model predicting hearing impairment at age 76.

First, to test whether childhood cognitive ability was associated with hearing impairment in older age, we ran a series of hierarchical ordinal logistic regression analyses with hearing impairment at age 76 (Wave 3), as the outcome variable. We ran two iterations of this model. The baseline model included age 11 IQ, age in days at Wave 3, and sex. Model 2 was additionally adjusted for occupational social class, symptoms of anxiety and depression, smoking status, and history of diabetes, cardiovascular disease, stroke, or hypertension. This latter model tested whether any association between childhood cognitive ability and later-life hearing impairment was accounted for by demographic and/or health-related differences. We note that we could not directly test for the system integrity mechanism outlined in the introduction (that shared variance in hearing and cognitive abilities originates at an early stage in the life-course) as data on childhood hearing ability were not collected. Figure 1 provides a summary of the ordinal logistic regression model.[Fig-anchor fig1]

Next, we tested whether hearing impairment at age 76 is associated with general cognitive ability at the same age or change in general cognitive ability over the following 3 years, and whether any such associations are confounded by childhood cognitive ability. At each wave, participants completed 13 mental ability tests, which can be treated as indicators of a latent factor representing general cognitive ability. We chose to examine associations with general cognitive ability because system integrity theory is typically applied to individual differences at this general level (Deary et al., 2012). Furthermore, previous work examining associations between hearing and cognitive abilities indicates that hearing impairment is related to a lower level of and steeper decline in general cognitive ability in older age (Loughrey et al., 2018).

We first established the factor structure of the general cognitive ability variable at age 76 and 79 and then tested for strong measurement invariance across the two waves of testing. Results from these preliminary analyses are reported in the results section.

**Figure 2 fig2:**
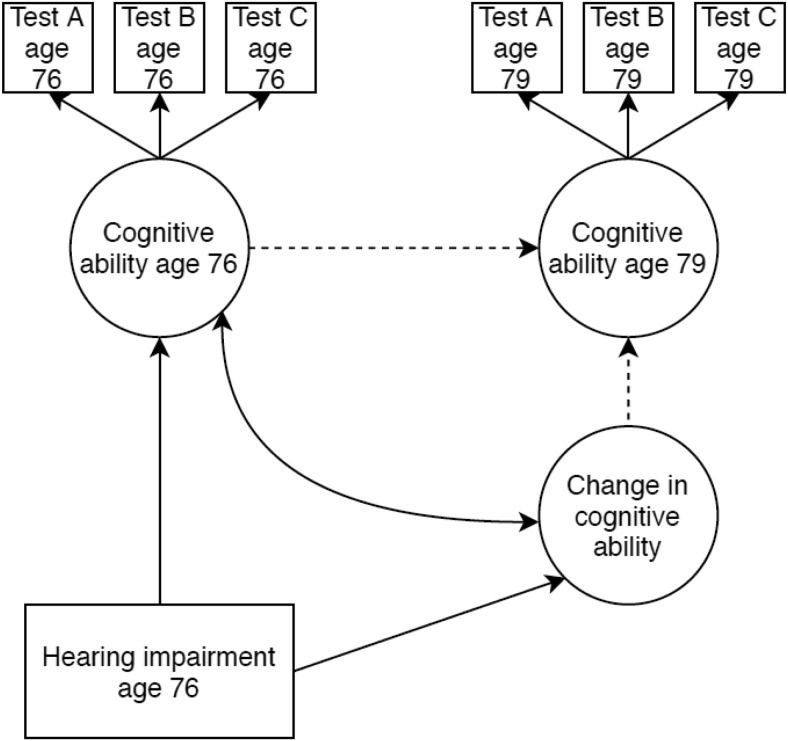
Simplified path diagram of the latent difference score model testing for associations between hearing ability at age 76 and level or change in cognitive ability. Ellipses represent latent variables, rectangles observed variables, double headed arrows correlations, and single headed arrows regression paths. Dotted lines indicate paths fixed to 1 for identification purposes. Only the first three cognitive ability tests are shown; however, 13 tests served as indicators of general cognitive ability at each wave. Also not shown, are correlations between measurement residuals of the cognitive ability tests. Measurement residuals of groups of tests from the same cognitive ability domain were allowed to correlate within and between waves (as described in the Method section).

The associations between older-age hearing impairment and general cognitive ability level and change were estimated with a latent change score model. This modeling framework is summarized by Newsom (2015) and shown in Figure 2. Briefly, the autoregressive path (from cognitive ability at age 76 to cognitive ability at age 79) was set equal to 1 as was the loading for the latent change score. Cognitive ability at age 76 was allowed to correlate with change in cognitive ability. We specified paths from the hearing impairment variable at age 76 (that was treated as an exogenous variable in this analysis) to cognitive ability at age 76 and change in cognitive ability between ages 76 and 79.[Fig-anchor fig2]

We adjusted for covariate variables at the manifest variable level, that is, the individual cognitive tests. This was achieved in three stages. First, cognitive test variables were adjusted for age in days at time of testing and sex (Model 1). Second, to test for the potentially confounding effect of childhood cognitive ability, cognitive test variables were additionally adjusted for age 11 IQ (Model 2). Finally, cognitive test variables were additionally adjusted for occupational social class, symptoms of anxiety and depression, smoking status, hearing aid use, and history of diabetes, cardiovascular disease, stroke, and hypertension (Model 3). Symptoms of anxiety and depression, smoking status, and history of chronic disease were assessed at ages 76 and 79, and treated as time varying covariates. Because we were interested in the effect of hearing impairment at age 76 in this analysis, we adjusted the cognitive test variables for hearing aid use at age 76 only.

**Figure 3 fig3:**
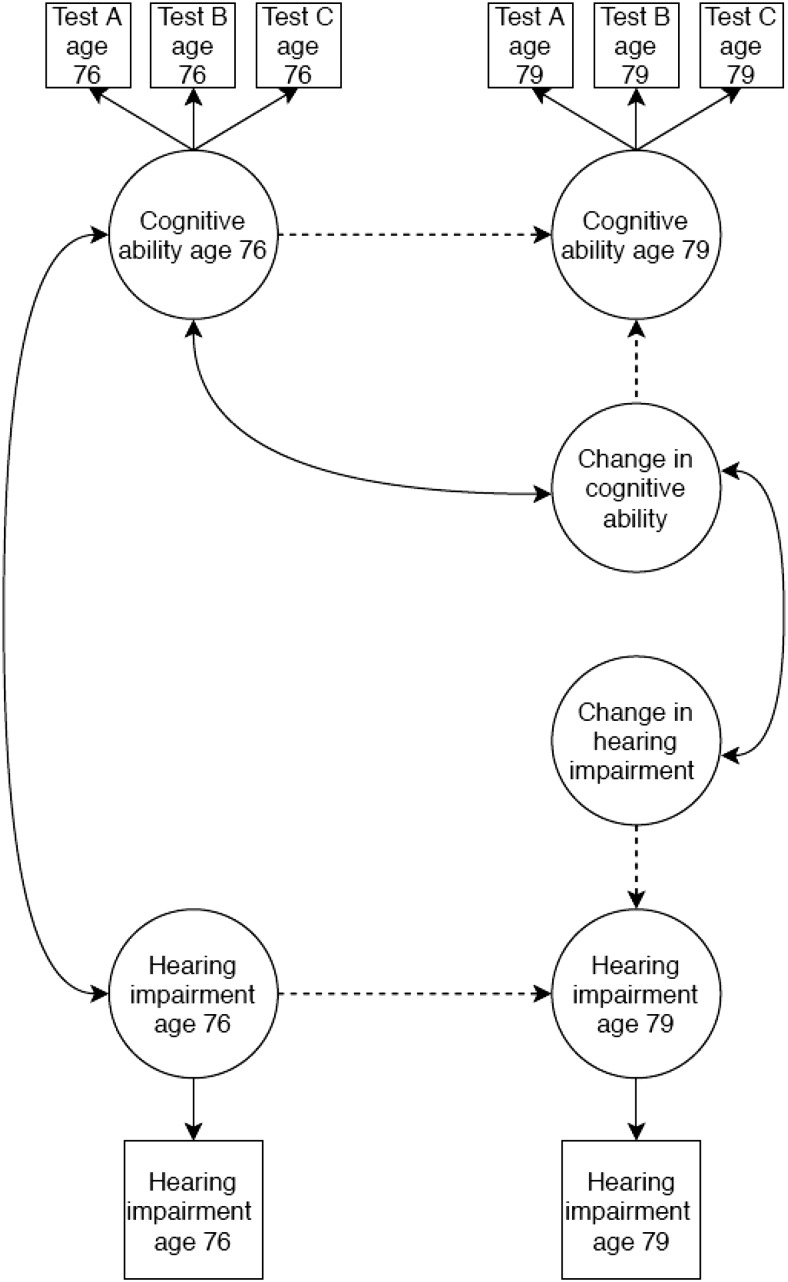
Simplified path diagram of the latent difference score model testing for associations between change in hearing ability and change in cognitive ability. The correlation between level and change variables cannot be estimated in latent change score models with categorical variables (Newsom, 2015); therefore, the correlation between hearing ability level and change was omitted. All other elements are as described in Figure 2.

We ran additional analyses to test whether change in hearing impairment was associated with change in cognitive function. This was achieved by additionally including hearing impairment at age 76 and 79 in the model and estimating a latent change score representing change in hearing impairment status. Hearing impairment was treated as an ordinal categorical variable in this analysis and the model was estimated using the weighted least square mean and variance adjusted (WLSMV) estimator and theta parametrization. The first threshold values for hearing impairment at age 76 and 79 were set equal to 0 (for identification purposes) and the second threshold values were held equal across the two waves. We allowed latent change scores for cognitive ability and hearing impairment to correlate. This model is summarized in Figure 3.[Fig-anchor fig3]

Full-information maximum likelihood estimation (FIML) was used to handle missing data at age 76 or 79. FIML estimates model parameters based on all available data, and is superior to other missing data strategies such as listwise deletion or mean imputation (Enders, 2001). Data are assumed to be missing at random, meaning that patterns of missingness are systematic and can be predicted by the observed data (Garson, 2015).

Finally, we corrected *p* values for multiple comparisons using Hochberg’s False Discovery Rate (FDR) correction (Benjamini & Hochberg, 1995). This correction was carried out separately for groups of *p* values considered to be from separate families of tests. Iterations of the same model, that is, including additional covariates, were considered as the same family of tests.

## Results

### Descriptive Data

Table 1 shows characteristics of the sample at ages 76 and 79. Mean scores on most of the cognitive tests decreased between waves, though not the crystallized ability tests. The proportion of participants reporting a history of diabetes, stroke, or hypertension increased between waves. At age 76, the number of participants with no hearing impairment, mild hearing impairment, and moderate/severe hearing impairment was 276 (40.1%), 303 (44.0%), and 110 (16.0%), respectively. Within the hearing impaired groups, the percentage of participants who reported wearing a hearing aid was 64.5% for moderate/severe hearing impairment and 16.5% for mild hearing impairment. Cohen’s unweighted κ statistic was calculated to determine test–retest reliability of the hearing impairment variable across the two assessment occasions at age 76 and age 79 (note that some change in hearing impairment status was expected over this 3-year period as risk of hearing impairment increases with older age). There was moderate agreement between the two hearing impairment assessments (κ = .555, *p* < .001). There was no association between hearing impairment and visual acuity at age 76 (*r*_s_ = .028, *p* = .482) or age 79 (*r*_s_ = .039, *p* = .392).

Table 2 shows correlations at age 76 among general cognitive ability, hearing impairment, demographic and health variables, and IQ at age 11. Table 3 shows the same correlations at age 79. Age 11 IQ was strongly correlated with general cognitive ability at age 76, *r* = .537, *p* < .001 and age 79 (*r* = .536, *p* < .001). Such associations between age 11 IQ and general cognitive ability in older-age have been documented previously in the LBC1936 (see e.g., Deary, Whalley, et al., 2009). Online supplemental material Tables 2 and 3 additionally show correlations among individual cognitive ability test scores and hearing impairment categories at ages 76 and 79. At age 76, hearing impairment was most strongly correlated with the NART and WTAR tests.[Table-anchor tbl2][Table-anchor tbl3]

### Childhood IQ and Hearing Impairment Risk

**Figure 4 fig4:**
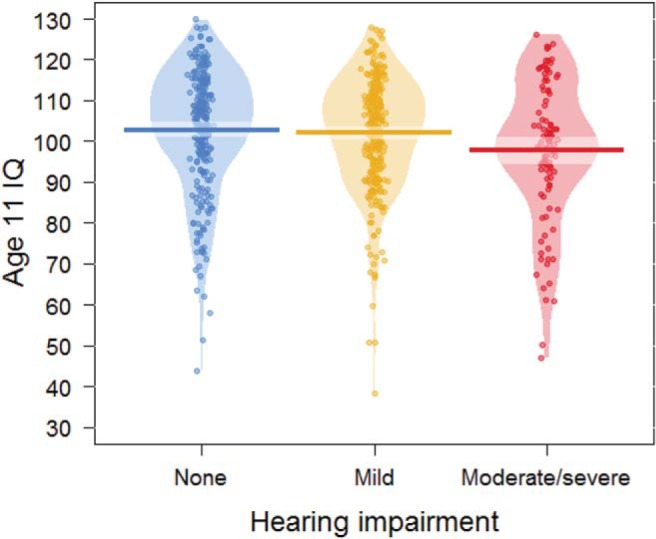
Plot of age 11 IQ by category of hearing impairment at age 76. Bars indicate mean scores; the lighter bands represent Bayesian highest density intervals. The “bean” shapes are smoothed density curves that show the full distribution of the data.

Figure 4 shows the relationship between age 11 IQ and hearing impairment at age 76. In Model 1 of the ordinal logistic regression (that controlled for sex and age in days at Wave 3) a higher age 11 IQ score was associated with lower odds of hearing impairment at age 76: odds ratio (*OR*) for being in a more severe hearing impairment category according to a *SD* increase in IQ = .834, 95% CI [.717, .970], FDR corrected *p* = .042. The association between higher age 11 IQ and lower odds of hearing impairment was nonsignificant after adjustment for additional covariates in Model 2 (occupational social class, symptoms of anxiety and depression, smoking status, and history of diabetes, cardiovascular disease, stroke, or hypertension), *OR* = .956, 95% CI [.809, 1.129], FDR *p* = .585.[Fig-anchor fig4]

### Hearing Impairment and General Cognitive Ability

#### Measurement model

Before running the latent change score model, we first established that the same latent structure of general cognitive ability existed at ages 76 and 79 by running separate cross-sectional confirmatory factor analysis for this variable at each age. General cognitive ability was scaled using the marker variable method, with the first cognitive test loading set to 1. Fit indices for these two models were initially poor. As documented previously in the LBC1936 (Ritchie et al., 2016), general cognitive ability in this sample is best characterized by a hierarchical structure whereby general cognitive function loads onto four second-order factors representing the cognitive ability domains of visuospatial ability, crystallized ability, verbal memory, and processing speed. This domain specific variance can be expressed in terms of correlated measurement errors (Gerbing & Anderson, 1984). Thus, measurement errors of variables in the same cognitive domain were allowed to correlate. This modification resulted in acceptable model fit for both age 76 and age 79 models (see online supplemental material Table 3). Matrix Reasoning, Block Design, and Spatial Span tests were treated as indicators of visuospatial ability; NART, WTAR, and Verbal Fluency tests were treated as indicators of crystallized ability; Verbal Paired Associates, Logical Memory, and Digit Span Backward tests were treated as indicators of verbal memory; and Symbol Search, Digit Symbol, Inspection Time, and Reaction Time tests were treated as indicators of processing speed. Standardized factor loadings for general cognitive ability at age 76 and 79 are displayed in Supplemental Table 4.

We also calculated the internal consistency of the general cognitive ability variable (as indicated by the 13 cognitive ability tests) at ages 76 and 79. McDonald’s ω (McDonald, 1999) showed good internal consistency at age 76 (ω = .805) and age 79 (ω = .817).

Next, in a longitudinal model of general cognitive ability at age 76 and 79, we established strong measurement invariance by imposing equality constraints on the loadings and intercepts of each repeated cognitive ability test. Measurement residuals of tests from the same cognitive ability domain were allowed to correlate across the two waves. Following the recommendation of Little (2013) we used change in comparative fit index (CFI) as an indicator of model fit. If change in CFI is not more than .01 then the assumption of invariance is acceptable (Cheung & Rensvold, 2002). An unconstrained model with all loadings freely estimated (with the exception of the first indicator) fit the data well, CFI = .969, root mean square error of approximation (RMSEA) = .046. Constraining factor loadings to be equal did not result in a worse fitting model (change in CFI = .001), suggesting that longitudinal metric invariance was met. Additionally constraining the intercepts to be equal over time did result in a worse fitting model (CFI = .946, RMSEA = .058; change in CFI = .022). However, RMSEA and CFI/Tucker-Lewis index (TLI) fit indices were still within the acceptable range: the CI for the RMSEA included .05, and CFI and TLI were >.90 (Hu & Bentler, 1999). Results from these model comparisons and additional fit indices are shown in online supplemental material Table 5. Following the advice of Widaman, Ferrer, and Conger (2010), we used the model with strong factorial invariance (loadings and intercepts held equal across measurement occasions) in the subsequent latent change score analysis, but conducted sensitivity analysis comparing our results to those obtained from a model imposing partial invariance (only loadings held equal).

#### Latent change score model

Fit indices for the latent change score model of hearing impairment at age 76 and general cognitive ability level (at age 76) and change (between ages 76 and 79) were within the acceptable range: RMSEA = .044, CFI = .964, TLI = .949. Estimates for the association between hearing and cognitive abilities represent a *SD* change in cognitive ability level or change according to a unit increase in the hearing impairment variable (i.e., going up one hearing impairment category). In Model 1, which was adjusted for sex and age at time of testing, hearing impairment was associated with a lower general cognitive ability level, β = −.119, 95% CI [−.203, −.035]; this association was maintained after correction for multiple comparisons, correcting for comparisons made in Models 1, 2, and 3 (FDR *p* = .030). The association between hearing impairment and cognitive ability level became nonsignificant in Model 2 that additionally adjusted for age 11 IQ (β = −.068, FDR *p* = .426) and remained nonsignificant in Model 3, which additionally adjusted for occupational social class, symptoms of anxiety and depression, smoking status, hearing aid use, and history of chronic disease (β = −.067, FDR *p* = .432). We found no evidence of an association between hearing impairment at age 76 and change in general cognitive ability between ages 76 and 79 in Model 1 (*r* = .078, FDR *p* = .842); Model 2 (β = −.002, FDR *p* = .981); or Model 3 (β = −.038, FDR *p* = .842).

We tested whether change in hearing impairment status between ages 76 and 79 was associated with change in general cognitive ability over the same time period (as outlined in Figure 3). In a model adjusted for sex and age in days at time of testing, change in hearing impairment status was not significantly related to change in general cognitive ability (*r* = .652, FDR *p* = .388). Therefore, we did not run further versions of this model, adjusting for age 11 IQ or other covariate variables.

### Sensitivity Analysis

We excluded participants with possible cognitive impairment from analysis predicting cognitive ability level and change. To test whether hearing-cognition associations were stronger in those participants, we reran our analysis also including participants with an MMSE score of less than 24. Results from this sensitivity analysis, which involved a sample of 696 participants, are displayed in online supplemental material Table 6. Estimates from this analysis were slightly larger than those obtained with the original sample. The association between hearing impairment and general cognitive ability level was just significant in Model 2 (following adjustment for age 11 IQ; β = −.090, *p* = .043); although this association did not survive correction for multiple comparisons (FDR *p* = .120).

Because the model imposing strong factorial invariance (loadings and intercepts of the cognitive ability tests held equal across measurement occasions) provided a worse fit to the data, according to CFI estimates, we reran the latent change score analysis imposing only partial factorial invariance (with only loadings held equal). Estimates from this sensitivity analysis were very similar to those obtained from the strong factorial invariance model and are displayed in online supplemental material Table 7.

Whereas crystallized abilities remain stable in older age, other more fluid cognitive abilities tend to decline. To test whether inclusion of the crystallized ability tests masked any association between hearing impairment and declining fluid cognitive abilities, we reran the latent change score analysis not including crystallized ability tests (NART, WTAR, and verbal fluency) as indicators of general cognitive ability. Results from this analysis are shown in online supplemental material Table 8. Effect sizes for the association between hearing impairment and cognitive ability level were slightly smaller than those obtained in the original analysis and the association between hearing impairment and cognitive ability level in Model 1 did not survive correction for multiple comparisons (β = −.105, *p* = .016, FDR *p* = .096). The association between hearing impairment and cognitive ability change remained nonsignificant in the minimally adjusted model (Model 1) and in the models additionally adjusting for age 11 IQ (Model 2) and occupational social class, symptoms of anxiety and depression, smoking status, hearing aid use, and history of chronic disease (Model 3).

In subsidiary analysis, we tested whether cognitive ability—IQ at age 11 or general cognitive ability at age 76—was associated with hearing aid use among participants with a hearing impairment (mild or moderate/severe) at age 76 (*n* = 397); this analytical sample excluded participants with possible cognitive impairment. In models adjusting for age and sex, neither age 11 IQ nor general cognitive ability at age 76 was associated with hearing aid use at age 76. Estimates for these associations were β = −.017, *p* = .800; and β = .043, *p* = .553, respectively.

Finally, we tested whether age 11 IQ moderated the association between hearing impairment and general cognitive ability by including an interaction effect between age 11 IQ and hearing impairment in the latent change score model predicting general cognitive ability level and change (additionally adjusting for age in days at time of testing and sex). The interaction effect was nonsignificant for cognitive ability level (FDR *p* = .456) and change (FRD *p* = .096).

## Discussion

It has been suggested that older people with poorer hearing are likely to perform less well on cognitive tests and experience more rapid cognitive decline (Loughrey et al., 2018). Here, we hypothesized that any such associations might be accounted for by childhood cognitive ability. To this end, we first tested whether cognitive ability measured in childhood is related to hearing impairment in later life. We found that having a higher cognitive ability at age 11 was associated with a slightly lower risk of hearing impairment at age 76, *OR* = .834. Next, we tested whether this association was mediated by occupational social class, symptoms of anxiety and depression, smoking status, and history of chronic disease, by additionally controlling for these variables in the model. We found that the association between childhood cognitive ability and older-age hearing impairment risk was reduced (*OR* = .956) and nonsignificant once potentially mediating variables were taken into account. Finally, we tested whether childhood cognitive ability contributes to any associations between hearing impairment and general cognitive ability level or change in older age. In our sample, hearing impairment at age 76 was related to lower general cognitive ability level at the same age (β = −.119) but not decline over the following 3 years. We tested for the potential role of childhood cognitive ability by additionally adjusting for this variable in the model. The cross-sectional association between hearing impairment and general cognitive ability at age 76 was reduced and nonsignificant (β = −.068) once variance related to childhood cognitive ability was accounted for, suggesting that this variable might account in part for associations between levels of older-age auditory and cognitive function. However, it is not clear from our study whether childhood cognitive ability contributes to associations between hearing impairment and age-related cognitive decline.

To our knowledge, this is the first study to document an association between cognitive ability in childhood and hearing ability in older age. This effect became nonsignificant once additional variables associated with risk of hearing impairment were taken into account. Thus, our findings suggest that a higher cognitive ability in childhood is associated with a lower risk of hearing impairment in older age, and that this association, in turn, can be accounted for in part by demographic and health-related differences. Risk factors (measured at age 76) significantly associated with childhood cognitive ability in our sample were occupational social class, HADS score, and history of diabetes. Occupational noise exposure (an established risk factor for hearing impairment), which is more prevalent in lower social class occupations (Lie et al., 2016), may also play a mediating role between childhood intelligence and later-life hearing impairment (although, such an effect may be less pronounced in younger cohorts), as work place noise exposure has been reduced in industrialized countries in recent decades (Lie et al., 2016).

In line with system integrity theory, it is possible that the association between childhood cognitive ability and older-age hearing impairment risk, observed in our study, also reflects lifelong shared variance between hearing and cognitive abilities. However, this hypothesis could not be tested fully, as data on childhood hearing ability were not collected. This possibility is relevant to life span developmental models of ageing which emphasize the processes of continuity and discontinuity (Baltes, Reese, & Lipsitt, 1980). Whereas “continuity” describes the moderate-to-strong stability of individual differences in certain traits (such as cognitive function) across the life-course, “discontinuity” describes changes that emerge as a result of factors unique to a specific phase in life, such as older age. Baltes and Lindenberger (1997) suggest that associations between sensory (hearing and vision) and cognitive function may emerge most strongly in older age (reflecting a process of developmental discontinuity) potentially as a result of age-related brain pathology affecting both cognitive and sensory functioning (consistent with the common cause hypothesis). Further longitudinal work tracking hearing and cognition across different phases of the life-course would help to clarify when associations are first established and how they change. We note that levels of hearing and visual acuity at ages 76 and 79 were not correlated in our study; this finding does not support the prediction, made by system integrity theory, of correlated intercepts across different bodily systems. We did not have data to test whether such associations existed earlier in the life course.

Our finding of a significant cross-sectional association between hearing impairment and lower general cognitive ability at age 76 is in line with results reported by previous studies (Baltes & Lindenberger, 1997; Gussekloo, de Craen, Oduber, van Boxtel, & Westendorp, 2005; Harrison Bush, Lister, Lin, Betz, & Edwards, 2015; Lin et al., 2011; Sugawara et al., 2011). The magnitude of this association was reduced and nonsignificant once childhood cognitive ability was accounted for in the model (this was the case in both analysis including and excluding participants with possible cognitive impairment). This result raises questions regarding the potential direction of effect between hearing and cognitive abilities. According to the sensory deprivation and effortfulness hypotheses, hearing impairment negatively impacts cognitive function in older age (either through reduced cognitive stimulation or reallocation of cognitive resources to the processing of auditory information). However, the attenuating effect of childhood cognitive ability on the association between older-age hearing and cognitive abilities, highlights the potential role of reverse causation: from earlier cognitive ability to later-life hearing impairment. As described in the paragraph above, this pathway appears to be mediated by health behaviors and environmental exposures associated with cognitive ability and relevant to hearing impairment risk.

The cross-sectional association between older-age hearing and cognitive ability was slightly stronger once participants with a low MMSE score were included in the analytical sample—suggesting that hearing impairment may be more closely associated with pathological cognitive ageing (although, because of the small number of participants with low MMSE scores we did not test this formally). A stronger association in those experiencing pathological cognitive decline would be in line with the findings of Deal et al. (2017) who report an association between hearing impairment and dementia risk, but no association between hearing impairment and cognitive decline in a healthy subset of participants without dementia. Higher childhood intelligence is related to lower dementia risk in women (Russ et al., 2017); thus, future research should test whether premorbid cognitive ability confounds associations between older-age hearing impairment and dementia risk.

In the present study, we focused on whether premorbid cognitive ability confounds the later-life association between auditory and cognitive abilities. However, premorbid cognitive ability may interact with these variables in other ways too. For instance (presuming hearing impairment and cognitive decline are causally related), childhood IQ may serve as a cognitive reserve factor, with higher IQ individuals being more resilient to the negative impact of hearing impairment on cognitive function. One previous study tested for this potential mechanism, with the prediction that cognitive reserve factors (as indexed by crystallized ability tests) might mask the association between hearing impairment and cognitive decline (Bucks et al., 2016). However, the authors found no association between older-age hearing and cognitive abilities in a model controlling for premorbid cognitive function and other covariate variables (education, depression, and sex). Similarly, in our study, we did not find evidence of an interaction between age 11 IQ and hearing impairment status in predicting general cognitive ability level at age 76 or cognitive decline over the subsequent 3 years.

Whereas hearing impairment was related to poorer cognitive function cross-sectionally, we failed to find evidence of a link between hearing impairment level (or hearing impairment change) and change in cognitive function between ages 76 and 79. This null result is surprising considering previous reports of a significant association between hearing impairment and cognitive decline (Deal et al., 2015; Gallacher et al., 2012; Lin et al., 2013). The prevalence of moderate/severe hearing impairment was low in our sample, potentially reflecting the association between hearing impairment and mortality risk (Fisher, Snih, Ostir, & Goodwin, 2004; Karpa et al., 2010). Although attrition between Waves 3 (age 76) and 4 (age 79) was not related to hearing impairment at age 76, it is possible that participants who left the study before age 76 had poorer hearing. In addition, participants with lower cognitive ability scores at age 76 were less likely to take part in the study at age 79. Both factors, low prevalence of hearing impairment and selective attrition related to cognitive ability, may have resulted in an underestimate of the association between hearing impairment and cognitive change in our study.

Other limitations of our study include the hearing impairment variable that was assessed at only two frequencies (1 and 3 kHz). Therefore, it is likely that we failed to identify participants with a hearing impairment at frequencies other than 1 and 3 kHz. In addition, we only assessed levels of pure-tone sensitivity using a simple hand-held device designed for screening, not accurate audiometry. Other methods of assessment, including tests of speech-in-noise hearing are more effective in identifying older adults with a functional hearing impairment, and may, therefore, be more sensitive to hearing-cognition associations, see Gates et al. (1996) for an illustration of this approach. Birth cohort effects should also be considered. There is evidence that younger generations maintain good hearing and cognitive function for longer, potentially reflecting increased access to education and a reduction in the prevalence of hearing impairment risk factors such as noise exposure and smoking (Rönnlund, Nyberg, Bäckman, & Nilsson, 2005; Zhan et al., 2010). Thus, it is possible that the association between childhood cognitive function and later-life hearing impairment will be weaker in younger cohorts, particularly if this association is mediated by health behaviors or environmental exposures that have recently been reduced.

Strengths of our study should also be noted. First, most longitudinal studies into hearing impairment and cognitive ability in older age used samples with relatively wide age ranges (with *SD*s in sample age ranging between 3 and 8 years; Loughrey et al., 2018). Analysis with such samples risks confounding by chronological age, and can yield inflated effect sizes (Deary et al., 2012; Hofer & Sliwinski, 2001). By testing for associations between hearing impairment and cognitive abilities in a narrow age cohort, we were able to minimize the risk of this confounding effect. Additionally, general cognitive ability was assessed with 13 tests on two occasions and modeled as a latent factor. This is a notable improvement on previous studies, many of which relied on dementia screening tests (e.g., the MMSE) as indicators of general cognitive function (Loughrey et al., 2018). A final advantage is that we could control for the effect of premorbid cognitive ability (assessed at age 11) as well as a range of demographic and health-related variables.

## Conclusion

There is growing interest in the relationship between hearing impairment and older-age cognitive function. Our results highlight the value of examining this association from a life-course perspective. In our sample, we found that cognitive ability in childhood was related to both cognitive and auditory function in older-age, and that childhood cognitive ability accounted for the cross-sectional association between auditory and cognitive ability in older age (reducing the effect size for this association by almost half). These results challenge the view that the association between cognitive and hearing ability originates in older age, and point to a potentially causal pathway from early life cognitive ability to later-life hearing impairment risk (via demographic and health-related factors). Further work is needed to test whether early life cognitive ability also contributes to associations between older-age hearing impairment and cognitive decline or dementia risk.

## Supplementary Material

10.1037/pag0000385.supp

## Figures and Tables

**Table 1 tbl1:** Sample Characteristics at Ages 76 and 79

Variable	Age 76 all	*n*	Age 76 completers	Age 79	*n*	*p*
Matrix reasoning	13.05 (4.90)	688	13.35 (4.90)	12.98 (5.01)	523	.017
Block design	32.21 (9.93)	690	32.76 (9.75)	31.34 (9.66)	523	<.001
Spatial span	14.63 (2.71)	689	14.83 (2.67)	14.16 (2.72)	524	<.001
Paired associates	26.41 (9.56)	663	27.29 (9.25)	27.31 (9.44)	487	.055
Logical memory	74.63 (19.17)	687	75.84 (18.41)	73.07 (20.29)	530	<.001
Digit span	7.77 (2.37)	694	7.91 (2.41)	7.58 (2.18)	536	<.001
NART	35.04 (8.02)	694	35.62 (7.94)	35.72 (8.10)	534	.427
WTAR	41.11 (7.01)	693	41.58 (6.91)	41.74 (6.97)	534	.135
Verbal fluency	42.91 (12.77)	695	43.78 (12.72)	43.75 (13.31)	535	.966
Digit symbol	53.85 (12.90)	684	55.48 (12.21)	51.44 (12.92)	523	<.001
Symbol search	24.62 (6.44)	686	25.28 (6.24)	22.72 (6.73)	517	<.001
Reaction time	.68 (.10)	684	.67 (.09)	.71 (.11)	531	<.001
Inspection time	110.14 (12.55)	654	110.97 (11.83)	107.05 (13.60)	458	<.001
Hearing impairment	689			525	<.001	
Not impaired	276 (40.1)		219 (41.2)	187 (35.6)		
Mild	303 (44.0)		229 (43.1)	232 (44.2)		
Moderate/severe	110 (16.0)		83 (15.4)	106 (20.2)		
Smoking status		695			538	.123
Nonsmoker	358 (51.5)		297 (55.2)	288 (53.5)		
Ex-smoker	293 (42.2)		222 (41.3)	229 (42.6)		
Smoker	44 (6.3)		19 (3.5)	21 (3.9)		
Diabetes	82 (11.8)	696	59 (11.0)	69 (12.9)	536	.003
CVD	235 (33.8)	695	184 (34.2)	197 (36.7)	537	.076
Stroke	73 (10.5)	696	55 (10.2)	67 (12.5)	535	.023
Hypertension	377 (54.2)	695	285 (53.0)	313 (58.2)	538	<.001
HADS	7.56 (4.51)	695	7.36 (4.45)	7.03 (4.46)	536	.026
Age	76.25 (.68)	696	76.24 (.67)	79.31 (.62)	538	
Follow-up years				3.08 (.28)	538	
Use hearing aid	136 (19.5)	696	113 (21.0)			
Female	336 (48.3)	696	266 (49.4)			
Age 11 IQ	101.62 (15.16)	654	102.10 (14.99)			
HMSO social class	687					
Professional	142 (20.7)		124 (23.4)			
Managerial	266 (38.7)		208 (39.3)			
Nonmanual	141 (20.5)		103 (19.5)			
Skilled manual	110 (16.0)		75 (14.2)			
Partly/unskilled	28 (4.0)		19 (3.6)			
*Note*. NART = National Adult Reading Test; WTAR = The Wechsler Test of Adult Reading; HADS = Hospital Anxiety and Depression Score; HMSO social class = Her Majesty’s Stationery Office social class. Data are shown as mean (*SD*) or *n* (%). *p* is for within-participant change in cognitive test scores between age 76 and 79.						

**Table 2 tbl2:** Correlations at Age 76 Among General Cognitive Ability, Hearing Impairment, Demographic and Health Variables, and IQ at Age 11

Variable	1	2	3	4	5	6	7	8	9	10	11
1. General cognitive ability	—										
2. Hearing impairment	−.095*	—									
3. Age 11 IQ	.537**	−.096*	—								
4. HMSO social class	.321**	−.121**	.390**	—							
5. Smoking status	−.069	.005	−.068	−.036	—						
6. Diabetes	−.106**	.117**	−.096*	−.020	.057	—					
7. CVD	−.101**	.078*	−.049	.011	.079*	.092*	—				
8. Stroke	−.095*	.000	−.014	.023	.053	.212**	.060	—			
9. Hypertension	−.077*	.026	−.050	−.025	.013	.157**	.181**	.122**	—		
10. Sex	.014	−.101**	.090*	.159**	−.067	−.097*	−.134**	−.026	.026	—	
11. HADS	−.297**	.097*	−.157**	−.077*	.081*	.052	.032	.071	.078*	.104**	—
*Note*. HADS = Hospital Anxiety and Depression Score; HMSO social class = Her Majesty’s Stationery Office social class. Higher hearing impairment score = poorer hearing, higher social class score = higher social class; smoking status coded as 1 = never smoker, 2 = ex-smoker and 3 = current smoker; sex coded as 1 = male, 2 = female; higher HADS score = greater level of depression or anxiety. Estimates from analysis excluding participants with possible cognitive impairment. Pearson correlation coefficients are reported for correlations between continuous variables and between continuous and dichotomous variables (Point-Biserial correlations). Spearman’s correlation coefficients are reported for correlations involving ordinal variables (hearing impairment, social class, and smoking status).											
* *p* < .05. ** *p* < .001.											

**Table 3 tbl3:** Correlations at Age 79 Among General Cognitive Ability, Hearing Impairment, Demographic and Health Variables, and IQ at Age 11

Variable	1	2	3	4	5	6	7	8	9	10	11
1. General cognitive ability	—										
2. Hearing impairment	−.129**	—									
3. Age 11 IQ	.536**	−.066	—								
4. HMSO social class	.320**	−.122**	.390**	—							
5. Smoking status	−.055	−.002	−.074	−.001	—						
6. Diabetes	−.093*	.076	−.093*	.000	.091	—					
7. CVD	−.092*	.069	−.062	.013	.133**	.003	—				
8. Stroke	−.085	.049	−.045	.044	.027	.120**	−.027	—			
9. Hypertension	−.051	.028	−.032	.008	.054	.148**	.123**	.167**	—		
10. Sex	.017	−.095*	.090*	.159**	−.062	−.060	−.107*	−.040	−.001	—	
11. HADS	−.189**	.070	−.094*	−.057	.019	.006	.048	.008	.075	.080	—
*Note.* HADS = Hospital Anxiety and Depression Score; HMSO social class = Her Majesty’s Stationery Office social class. Higher hearing impairment score = poorer hearing, higher social class score = higher social class; smoking status coded as 1 = never smoker, 2 = ex-smoker and 3 = current smoker; sex coded as 1 = male, 2 = female; higher HADS score = greater level of depression or anxiety. Estimates from analysis excluding participants with possible cognitive impairment. Pearson correlation coefficients are reported for correlations between continuous variables and between continuous and dichotomous variables (Point-Biserial correlations). Spearman’s correlation coefficients are reported for correlations involving ordinal variables (hearing impairment, social class, and smoking status).											
* *p* < .05. ** *p* < .001.
