# Supplementary material for: Longitudinal Associations Between Hearing Loss and General Cognitive Ability: The Lothian Birth Cohort 1936
Source: Psychol Aging. 2019 Aug 8;34(6):766–79. doi: 10.1037/pag0000385 (PMC6742482; doi:10.1037/pag0000385)
Supplement: Supplementary file 1 [file PAG-2018-0247_Suppl.docx]

**Supplementary tables**

| Supplementary table 1  *Differences at age 76 between participants who provided data at ages 76 and 79 (‘completers’) and participants who provided data at age 76 only (‘non-completers’)* | | | | | |
| --- | --- | --- | --- | --- | --- |
| Characteristics at age 76 | Completers  total *n* = 538 | *n* | Non-completers  total *n* = 158 | *n* | *p* |
| Matrix reasoning | 13.35 (4.90) | 531 | 12.04 (4.79) | 157 | .003 |
| Block design | 32.76 (9.75) | 533 | 30.36 (10.32) | 157 | .010 |
| Spatial span | 14.83 (2.67) | 532 | 13.95 (2.76) | 157 | .001 |
| Paired associates | 27.29 (9.25) | 515 | 23.35 (10.00) | 148 | <.001 |
| Logical memory | 75.84 (18.41) | 534 | 70.37 (21.15) | 153 | .004 |
| Digit span | 7.91 (2.41) | 536 | 7.30 (2.20) | 158 | .003 |
| NART | 35.62 (7.94) | 536 | 33.08 (8.00) | 158 | .001 |
| WTAR | 41.58 (6.91) | 536 | 39.49 (7.13) | 157 | .001 |
| Verbal fluency | 43.78 (12.72) | 537 | 39.98 (12.54) | 158 | .001 |
| Digit symbol | 55.48 (12.21) | 526 | 48.41 (13.65) | 158 | <.001 |
| Symbol search | 25.28 (6.24) | 530 | 22.38 (6.63) | 156 | <.001 |
| Reaction time | 0.67 (0.09) | 527 | 0.71 (0.121) | 157 | <.001 |
| Inspection time | 110.97 (11.83) | 516 | 107.01 (14.58) | 138 | .004 |
| Hearing impairment |  | 531 |  | 158 | .303 |
| Not impaired | 219 (41.2) |  | 57 (36.1) |  |  |
| Mild impairment | 229 (43.1) |  | 74 (46.8) |  |  |
| Moderate/Severe impairment | 83 (15.4) |  | 27 (17.1) |  |  |
| Female | 266 (49.4) | 538 | 70 (44.3) | 158 | .256 |
| Smoking status |  | 538 |  | 157 | <.001 |
| Non-smoker | 297 (55.2) |  | 60 (38.9) |  |  |
| Ex-smoker | 222 (41.3) |  | 71 (44.9) |  |  |
| Smoker | 19 (3.5) |  | 25 (15.9) |  |  |
| HMSO social class |  | 529 |  | 158 | .006 |
| Professional | 124 (23.4) |  | 18 (11.4) |  |  |
| Managerial and Technical | 208 (39.3) |  | 58 (36.7) |  |  |
| Skilled non-manual | 103 (19.5) |  | 38 (24.1) |  |  |
| Skilled manual | 75 (14.2) |  | 35 (22.2) |  |  |
| Partly skilled or unskilled | 19 (3.6) |  | 7 (4.4) |  |  |
| Diabetes | 59 (11.0) | 538 | 23 (14.6) | 158 | .218 |
| Cardiovascular disease | 184 (34.2) | 538 | 51 (32.5) | 157 | .689 |
| Stroke | 55 (10.2) | 538 | 18 (11.4) | 158 | .673 |
| Hypertension | 285 (53.0) | 538 | 92 (58.2) | 157 | .213 |
| Age 11 IQ | 102.10 (14.99) | 507 | 99.35 (15.57) | 147 | .045 |
| HADS | 7.36 (4.45) | 538 | 8.22 (4.67) | 157 | .041 |

| Supplementary table 2.1  *Correlations among cognitive test scores and hearing impairment at age 76* | | | | | | | | | | | | | | |
| --- | --- | --- | --- | --- | --- | --- | --- | --- | --- | --- | --- | --- | --- | --- |
| Variable | 1 | 2 | 3 | 4 | 5 | 6 | 7 | 8 | 9 | 10 | 11 | 12 | 13 | 14 |
| 1. Age 11 IQ | - |  |  |  |  |  |  |  |  |  |  |  |  |  |
| 2. Matrix reasoning | .376^**^ | - |  |  |  |  |  |  |  |  |  |  |  |  |
| 3. Block design | .399^**^ | .554^**^ | - |  |  |  |  |  |  |  |  |  |  |  |
| 4. Spatial span | .216^**^ | .394^**^ | .422^**^ | - |  |  |  |  |  |  |  |  |  |  |
| 5. Verbal pairs | .285^**^ | .288^**^ | .162^**^ | .152^**^ | - |  |  |  |  |  |  |  |  |  |
| 6. Logical memory | .352^**^ | .281^**^ | .240^**^ | .213^**^ | .505^**^ | - |  |  |  |  |  |  |  |  |
| 7. Digit backwards | .380^**^ | .371^**^ | .290^**^ | .299^**^ | .297^**^ | .301^**^ | - |  |  |  |  |  |  |  |
| 8. NART | .680^**^ | .404^**^ | .375^**^ | .149^**^ | .394^**^ | .410^**^ | .404^**^ | - |  |  |  |  |  |  |
| 9. WTAR | .655^**^ | .375^**^ | .343^**^ | .143^**^ | .374^**^ | .386^**^ | .418^**^ | .890^**^ | - |  |  |  |  |  |
| 10. Verbal fluency | .400^**^ | .228^**^ | .208^**^ | .164^**^ | .331^**^ | .237^**^ | .334^**^ | .441^**^ | .436^**^ | - |  |  |  |  |
| 11. Digit symbol | .398^**^ | .372^**^ | .407^**^ | .282^**^ | .255^**^ | .365^**^ | .313^**^ | .379^**^ | .342^**^ | .386^**^ | - |  |  |  |
| 12. Symbol search | .386^**^ | .422^**^ | .503^**^ | .372^**^ | .225^**^ | .302^**^ | .331^**^ | .346^**^ | .327^**^ | .306^**^ | .637^**^ | - |  |  |
| 13. Reaction time | -.184^**^ | -.258^**^ | -.328^**^ | -.323^**^ | -.226^**^ | -.258^**^ | -.240^**^ | -.198^**^ | -.154^**^ | -.265^**^ | -.534^**^ | -.499^**^ | - |  |
| 14. Inspection time | .172^**^ | .287^**^ | .339^**^ | .251^**^ | .168^**^ | .160^**^ | .175^**^ | .176^**^ | .183^**^ | .245^**^ | .361^**^ | .403^**^ | -.355^**^ | - |
| 15. Hearing impairment | -.092^*^ | -.090^*^ | -.087^*^ | -.060 | -.050 | .057 | -.066 | -.129^**^ | -.119^**^ | -.059 | -.093^*^ | -.067 | .065 | -.006 |
| *Note.* ** *p* <0.001, **p* <0.05, higher score for hearing impairment = poorer hearing. Estimates from analysis excluding participants with possible cognitive impairment. NART = National Adult Reading Test, WTAR = The Wechsler Test of Adult Reading. | | | | | | | | | | | | | | |

| Supplementary table 2.2  *Correlations among cognitive test scores and hearing impairment at age 79* | | | | | | | | | | | | | | |
| --- | --- | --- | --- | --- | --- | --- | --- | --- | --- | --- | --- | --- | --- | --- |
| Variable | 1 | 2 | 3 | 4 | 5 | 6 | 7 | 8 | 9 | 10 | 11 | 12 | 13 | 14 |
| 1. Age 11 IQ |  |  |  |  |  |  |  |  |  |  |  |  |  |  |
| 2. Matrix reasoning | .421^**^ |  |  |  |  |  |  |  |  |  |  |  |  |  |
| 3. Block design | .378^**^ | .540^**^ |  |  |  |  |  |  |  |  |  |  |  |  |
| 4. Spatial span | .217^**^ | .385^**^ | .382^**^ |  |  |  |  |  |  |  |  |  |  |  |
| 5. Verbal pairs | .303^**^ | .326^**^ | .252^**^ | .123^**^ |  |  |  |  |  |  |  |  |  |  |
| 6. Logical memory | .330^**^ | .392^**^ | .234^**^ | .241^**^ | .538^**^ |  |  |  |  |  |  |  |  |  |
| 7. Digit backwards | .403^**^ | .352^**^ | .274^**^ | .311^**^ | .289^**^ | .307^**^ |  |  |  |  |  |  |  |  |
| 8. NART | .674^**^ | .410^**^ | .304^**^ | .151^**^ | .377^**^ | .360^**^ | .404^**^ |  |  |  |  |  |  |  |
| 9. WTAR | .639^**^ | .388^**^ | .297^**^ | .175^**^ | .352^**^ | .324^**^ | .408^**^ | .886^**^ |  |  |  |  |  |  |
| 10. Verbal fluency | .360^**^ | .265^**^ | .230^**^ | .181^**^ | .310^**^ | .245^**^ | .362^**^ | .368^**^ | .366^**^ |  |  |  |  |  |
| 11. Digit symbol | .386^**^ | .368^**^ | .382^**^ | .369^**^ | .292^**^ | .363^**^ | .375^**^ | .344^**^ | .308^**^ | .425^**^ |  |  |  |  |
| 12. Symbol search | .363^**^ | .441^**^ | .494^**^ | .385^**^ | .252^**^ | .335^**^ | .366^**^ | .329^**^ | .310^**^ | .352^**^ | .633^**^ |  |  |  |
| 13. Reaction time | -.276^**^ | -.296^**^ | -.302^**^ | -.313^**^ | -.237^**^ | -.273^**^ | -.264^**^ | -.205^**^ | -.156^**^ | -.344^**^ | -.594^**^ | -.442^**^ |  |  |
| 14. Inspection time | .145^**^ | .352^**^ | .307^**^ | .273^**^ | .199^**^ | .291^**^ | .155^**^ | .166^**^ | .159^**^ | .146^**^ | .401^**^ | .422^**^ | -.352^**^ |  |
| 15. Hearing impairment | -.084 | -.093^*^ | -.072 | -.064 | -.097^*^ | .005 | -.089^*^ | -.076 | -.105^*^ | -.108^*^ | -.109^*^ | -.066 | .115^**^ | -.082 |
| *Note.* ** *p* <0.001, **p* <0.05, higher score for hearing impairment = poorer hearing. Estimates from analysis excluding participants with possible cognitive impairment. NART = National Adult Reading Test; WTAR = The Wechsler Test of Adult Reading. | | | | | | | | | | | | | | |

| Supplementary table 3  *Changes in fit of cross-sectional confirmatory factor analysis models of general cognitive ability at age 76 and 79.* | | | | | | | |
| --- | --- | --- | --- | --- | --- | --- | --- |
| Model | X^2^ | *df* | CFI | BIC | RMSEA | X^2^ △ | *p* |
| Age 76 unstructured model | 1356 | 65 | 0.633 | 25,647 | 0.172 |  |  |
| Age 76 domain measurement errors correlated | 229 | 50 | 0.949 | 24,618 | 0.073 | -1127 | <.001 |
| Age 79 unstructured model | 1029 | 65 | 0.641 | 19,657 | 0.169 |  |  |
| Age 79 domain measurement errors correlated | 165 | 50 | 0.957 | 18,886 | 0.067 | -864 | <.001 |

*Note. df* = degrees of freedom, CFI = Comparative Fit Index, BIC = Bayesian Information Criterion, RMSEA = Root Mean Square Error of Approximation.

| Supplementary table 4  *Standardized factor loadings from cross-sectional confirmatory factor analysis* *for the general cognitive ability variable at ages 76 and 79* | | | | | | |
| --- | --- | --- | --- | --- | --- | --- |
|  |  | Age 76 | |  | Age 79 | |
| Cognitive test | Domain | Estimate | SE |  | Estimate | SE |
| Matrix Reasoning | Visuospatial ability | 0.611 | 0.032 |  | 0.640 | 0.034 |
| Block Design | Visuospatial ability | 0.582 | 0.035 |  | 0.555 | 0.040 |
| Spatial Span | Visuospatial ability | 0.420 | 0.040 |  | 0.466 | 0.043 |
| Verbal Pairs | Verbal memory | 0.477 | 0.039 |  | 0.503 | 0.043 |
| Logical Memory | Verbal memory | 0.523 | 0.036 |  | 0.540 | 0.039 |
| Digits Span Backwards | Verbal memory | 0.576 | 0.034 |  | 0.590 | 0.037 |
| NART | Crystallised ability | 0.643 | 0.032 |  | 0.576 | 0.038 |
| WTAR | Crystallised ability | 0.618 | 0.033 |  | 0.547 | 0.039 |
| Verbal Fluency | Crystallised ability | 0.501 | 0.037 |  | 0.527 | 0.039 |
| Digit Symbol | Processing speed | 0.630 | 0.032 |  | 0.657 | 0.034 |
| Symbol Search | Processing speed | 0.635 | 0.033 |  | 0.670 | 0.035 |
| Reaction Time | Processing speed | -0.443 | 0.039 |  | -0.494 | 0.042 |
| Inspection Time | Processing speed | 0.392 | 0.042 |  | 0.448 | 0.048 |
| *Note.* Sample size at Wave 3 = 673, and Wave 4 = 520; lower scores on the Reaction Time test = better performance. SE = standard error. | | | | | | |

| Supplementary table 5  *Fit indices for longitudinal models of general cognitive ability with and without equality constraints* | | | | | | | |
| --- | --- | --- | --- | --- | --- | --- | --- |
| Model | X^2^ | df | CFI | TLI | BIC | RMSEA | △CFI |
| Unconstrained | 548.994 | 225 | 0.969 | 0.956 | 39,423 | 0.046 |  |
| Loadings equal | 574.744 | 237 | 0.968 | 0.956 | 39,371 | 0.046 | 0.001 |
| Loadings equal and intercepts equal | 814.650 | 249 | 0.946 | 0.930 | 39,533 | 0.058 | 0.022 |

*Note. df* = degrees of freedom, CFI = Comparative Fit Index, BIC = Bayesian Information Criterion, RMSEA = Root Mean Square Error of Approximation

Sensitivity analysis 1

Including participants with MMSE <24

*n* = 696

| Supplementary table 6  *Results from the model of hearing impairment and cognitive ability level (at age 76) and change (between ages 76 and 79)* | | | | | | |
| --- | --- | --- | --- | --- | --- | --- |
|  | Model 1 | | Model 2 | | Model 3 | |
| Path | Estimate | *p*/FDR *p* | Estimate | *p*/FDR *p* | Estimate | *p*/FDR *p* |
| Level hearing 🡪 level cognitive | -0.140 | .001/.006 | -0.090 | .043/.120 | -0.098 | .060/.120 |
| Level hearing 🡪 change cognitive | -0.011 | .904/.904 | -0.050 | .478/.574 | -0.071 | .393/.574 |
| *Note.* Poorer hearing = higher score. Model 1 is adjusted for sex and age in days. Model 2 is additionally adjusted for age 11 IQ. Model 3 is additionally adjusted for occupational social class, symptoms of anxiety and depression, smoking status and history of chronic disease (diabetes, cardiovascular disease, stroke or hypertension), and hearing aid use. *p* is the uncorrected *p*-value. FDR *p* controls for the false discovery rate. | | | | | | |

Sensitivity analysis 2

Relaxing strong factorial invariance assumption

*n* = 673

| Supplementary table 7  *Results from the model of hearing impairment and cognitive ability level (at age 76) and change (between ages 76 and 79)* | | | | | | |
| --- | --- | --- | --- | --- | --- | --- |
|  | Model 1 | | Model 2 | | Model 3 | |
| Path | Estimate | *p*/FDR *p* | Estimate | *p*/FDR *p* | Estimate | *p*/FDR *p* |
| Level hearing 🡪 level cognitive | -0.121 | .005/.030 | -0.068 | .143/.429 | -0.067 | .221/.442 |
| Level hearing 🡪 change cognitive | 0.076 | .553/.830 | 0.000 | .998/.998 | -0.037 | .712/.854 |
| *Note.* Poorer hearing = higher score. Model 1 is adjusted for sex and age in days. Model 2 is additionally adjusted for age 11 IQ. Model 3 is additionally adjusted for occupational social class, symptoms of anxiety and depression, smoking status and history of chronic disease (diabetes, cardiovascular disease, stroke or hypertension), and hearing aid use. *p* is the uncorrected *p*-value. FDR *p* controls for the false discovery rate. | | | | | | |

Sensitivity analysis 3

Excluding crystallised ability tests

*n* = 673

| Supplementary table 8  *Results from the model of hearing impairment and cognitive ability level (at age 76) and change (between ages 76 and 79)* | | | | | | |
| --- | --- | --- | --- | --- | --- | --- |
|  | Model 1 | | Model 2 | | Model 3 | |
| Path | Estimate | *p*/FDR *p* | Estimate | *p*/FDR *p* | Estimate | *p*/FDR *p* |
| Level hearing 🡪 level cognitive | -0.105 | .016/.096 | -0.061 | .194/.582 | -0.056 | .308/.603 |
| Level hearing 🡪 change cognitive | -0.058 | .528/.634 | -0.038 | .668/.668 | -0.085 | .402/.603 |
| *Note.* Poorer hearing = higher score. Model 1 is adjusted for sex and age in days. Model 2 is additionally adjusted for age 11 IQ. Model 3 is additionally adjusted for occupational social class, symptoms of anxiety and depression, smoking status and history of chronic disease (diabetes, cardiovascular disease, stroke or hypertension), and hearing aid use. *p* is the uncorrected *p*-value. FDR *p* controls for the false discovery rate. | | | | | | |
